# Current and Future Cost Burden of Myocardial Infarction in Australia: Dynamic Multistate Markov Model

**DOI:** 10.1007/s11606-025-09423-8

**Published:** 2025-03-04

**Authors:** Tamrat Befekadu Abebe, Jenni Ilomaki, Adam Livori, J. Simon Bell, Jedidiah I. Morton, Zanfina Ademi

**Affiliations:** 1 Centre for Medicine Use and Safety, Faculty of Pharmacy and Pharmaceutical Sciences, Monash University, Melbourne, Australia; 2 School of Public Health and Preventive Medicine, Monash University, Melbourne, Australia; 3 Pharmacy Department, Grampians Health Ballarat, Melbourne, Australia; 4 Faculty of Health Sciences, University of Eastern Finland, Kuopio, Finland; 5 Baker Heart and Diabetes Institute, Melbourne, Australia; 6 Central Clinical School, Monash University, Melbourne, Australia

**Keywords:** cardiovascular disease, myocardial infarction, cost of illness, epidemiology, dynamic modelling

## Abstract

**Introduction:**

Myocardial infarction (MI) imposes a significant health burden to the Australian population. However, detailed economic implication of MI on the Australian healthcare system has not been exhaustively explored.

**Objective:**

To estimate the current chronic management cost and project the future healthcare cost burden of MI, from the Australian healthcare system perspective.

**Design:**

A generalized linear model with a gamma outcome distribution and negative inverse link function was used to estimate the current chronic management cost burden of MI while a dynamic multistate Markov model constructed to project the future healthcare cost burden of MI over 20 years (2019–2038). For all projected costs, 5% annual discounting was applied in the base case, as per Australian guidelines.

**Participants:**

We identified all people, 59,260, aged ≥ 30 years discharged from a public or private hospital following MI between 2012 and 2017 from the Victorian Admitted Episode Dataset. We estimated annual chronic management cost of MI by age, sex, socioeconomic disadvantage and years of follow-up. We used these data to project the future healthcare cost burden of MI.

**Main Measure:**

Cost in Australian dollar (AUD).

**Key Results:**

The current annual chronic management cost of MI was estimated to be AUD 14,412 (95% confidence interval: AUD 14,282, AUD 14,542) per person. This cost was higher among advanced age group, male participants, during first year of follow-up and people in the most socioeconomically disadvantaged quintile. The projected total healthcare cost following MI was AUD 85.1 billion (95% uncertainty interval AUD 80.8 billion, AUD 89.8 billion) from 2019 to 2038.

**Conclusion:**

Our projections suggest that MI will cost the Australian healthcare system over AUD 85 billion in the coming years. Cost estimates based on key sociodemographic characteristics and socioeconomic disadvantage are expected to inform future health economic modelling studies for MI prevention strategies and interventions.

**Supplementary Information:**

The online version contains supplementary material available at 10.1007/s11606-025-09423-8.

## INTRODUCTION

Ischemic heart disease was responsible for more than 9.4 million deaths and 185 million disability-adjusted life years globally in 2021.^1^ Acute coronary syndrome (ACS), comprising acute myocardial infarction (MI) and unstable angina, was responsible for 51,000 events in Australia in 2018, of which 40,200 were new events (34,000 non-fatal and 6200 fatal).^2^ The impact of MI extends beyond morbidity and mortality. MI is associated with profound economic costs for the individuals who experience MI and the healthcare system.^3–8^

The staggering cost impact of coronary heart disease (CHD) was reported by studies from European Union and the USA.^3,7^ In Australia, the Heart Foundation reported ACS would cost the Australian Government 1.9 billion Australian dollars (AUD) in 2017/2018.^5^ Furthermore, ACS accounted for AUD 3.7 billion in productivity loss, AUD 620.3 million in out-of-pocket costs and AUD 644.6 million in informal care costs.^4^ A recent study projected cardiovascular diseases (CVD) would cost the Australian healthcare system around AUD 61.9 billion between 2020 and 2029.^8^

Current Australian cost estimates are reported at the broad level—there is still a paucity of data related to current cost burden of MI by important sociodemographic characteristics including age, sex, comorbidities, socioeconomic disadvantage and year of follow-up.^4–6,8^ Moreover, previous Australian projection studies were based on the Australian National Health Survey which may lack granular clinical data on MI.^9,10^ Hence, the current study used a robust, large, linked data, which is capable of providing accurate and generalizable cost estimates for people with MI.

The overall objective of this study was to estimate the current and future healthcare cost burden of MI in Australia from a healthcare system perspective.

## METHOD

### Methods for Estimating the Current Cost Burden of Myocardial Infarction

#### Data Sources

The cohort for estimating the current chronic management cost of MI was identified from the VAED. We included people discharged from public or private hospitals across Victoria after admission due to MI (International Classification of Diseases (ICD)−10-Australian Modified (AM) codes: I21-I22) between 1 July 2012 and 30 June 2017 (with follow-up data until 30 June 2018).^11–14^ To estimate costs, the Australian Institute of Health and Welfare (AIHW) linked data from the VAED to data from the Pharmaceutical Benefits Scheme (PBS) related to medication dispensing (see Supplementary figure S1 for details) and to data from the Medicare Benefits Schedule (MBS) related to investigations and procedures (see Supplementary figure S2 for details).^11^ The AIHW also linked the cohort to National Death Index (NDI) to track vital status.^11^ Hospital admission costs were derived from the National Hospital Cost Data Collection (NHCDC) report covering the years 2012/13 to 2017/18 (see Supplementary Figure S3 for details). Our study adopted a healthcare system perspective, and all costs were adjusted to 2019 AUD using the Health Price Index^15^ to make the values consistent with the cost burden projection (which begun in 2019).

#### Exposures

We stratified the chronic management costs of MI by a number of demographic and clinical characteristics, including age, sex, type of MI (ST-elevation MI (STEMI; ICD-10-AM codes I210-I213) and non-STEMI (I214-I219)), comorbidities (hypertension and diabetes), socioeconomic disadvantage and duration of follow-up (i.e. time since the MI).^11^ Diabetes and hypertension status were defined via secondary diagnosis codes during admission with MI.^11,14^ The Australian Index of Relative Socioeconomic Disadvantage (IRSD) was used to summarize information about the economic and social condition within an area.^11,16^ We assigned an IRSD to each individual based on their last known postcode before admission for MI.^11^ Using the IRSD, areas were split into quintiles (relative to the entire population of Australia), the first quintile being the most socioeconomic disadvantaged and the fifth quintile being the least disadvantaged.^6,11,16^

### Statistical Analysis

A generalized linear model with a gamma outcome distribution and negative inverse link function, weighted by person-years of follow-up, was used to estimate cost. Univariable models were fit to estimate the overall chronic management cost by age (where age was the mid-point of each age group (in 5-year intervals) and parameterized with spline effects), type of MI, presence of comorbidity status (diabetes and hypertension), socioeconomic disadvantage (IRSD) and year of follow-up. Multivariable models were fitted to estimate the adjusted overall chronic management costs after adjusting for covariates listed above. The crude and adjusted chronic management cost were presented as annual cost per person with the respective 95% confidence intervals (CIs).

### Methods for Projecting the Future Cost Burden of Myocardial Infarction

#### Model Overview

A dynamic multistate Markov model was constructed to project the cost burden of MI for the Australian population aged 30 to 99 years, covering the period from 2019 to 2038 in annual cycles. The main outcome from the model was total healthcare costs—a combination of acute events cost of MI and chronic management cost following MI (i.e. any health-related cost after index MI).

#### Model Structure

**Figure 1:**
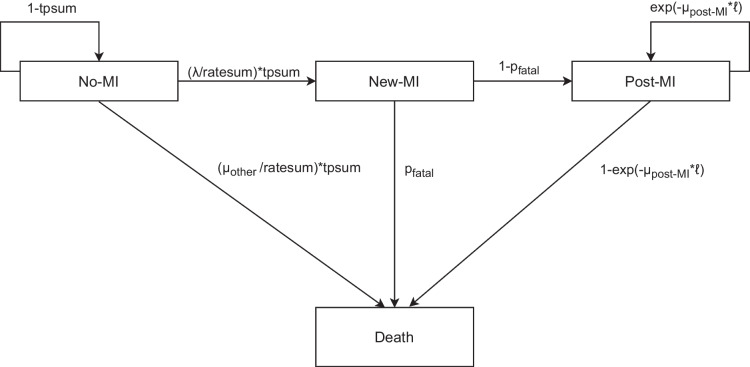
Structure of the multistate Markov model and associated transition probabilities. MI, myocardial infarction; ℓ: 1-year period, λ: myocardial infarction incidence rate, µ_other_: mortality rate for people without myocardial infarction (i.e. other mortality), µ_post-MI_: post-myocardial infarction all-cause mortality rate, p_fatal_: proportion of fatal myocardial infarction ratesum: λ + µ_other_; tpsum: 1-exp(-ratesum* ℓ). Ratesum is the sum of incidence rate of Myocardial infarction and mortality rate for people without myocardial infarction (i.e. other mortality); tpsum: transition probability of ratesum.

The core dynamic multistate Markov model (Fig. 1) was adopted from a previous Australian projection of health burden of MI for the period 2019 to 2038.^13^ Briefly, the model constitutes four health states including three alive health states and one death health state (the only absorbing health state). The alive health states were “alive, with no MI”, “alive, new MI” and “alive, post-MI”. The death health state could be due to “death, without MI (i.e. death other)”, “fatal-MI” or “death, post-MI”. Transitions between health states were simulated using a Markov model. The dynamic nature of the model reflects the consideration of demographic change over the course of projection time where people who were aged under 30 years in previous years could enter the model once they reach age 30.

#### Population

The Australian population aged 30 to 99 years in 2018 was used as the initial population for projection (see Supplementary Table S1).^17^ This population was then followed in annual cycles from 2019 to 2038. Australian residents and migrants were included in the model throughout the projection time whenever they were eligible to be part of the model (i.e. once they aged from 29 to 30 years at the beginning of each cycle or immigrated into Australia).^18^ The cohort exited the model under one of the three conditions: (a) after the 20-year follow-up; (b) upon reaching age 99, even if not been followed for the full 20 years; or (c) upon death before the end of follow-up period. The model assumed the age- and sex-specific rates and proportion remained constant throughout the projection period.

#### Data Sources

Data inputs to derive the transition probabilities for the dynamic model were primarily sourced from the VAED. Description of the VAED and data linkage was stated above.^11,13^ Victoria is the second populous state in Australia with a population of more than 6.5 million (25.6% of the total population) in 2021.^19^ Furthermore, Victoria has a similar age, sex, socioeconomic status, remoteness and ancestry distribution compared with the total Australian population.^19^ Thus, our sample is likely reasonably representative of Australia. In addition to the VAED, age-group and sex-specific prevalence of MI and all-cause mortality from the general Australian population was supplemented from the AIHW and Australian Bureau of Statistics (ABS), respectively.^20,21^

Costs incorporated in the dynamic model were based on the healthcare system perspective. Acute event costs related to hospitalization due to MI were sourced from the NHCDC version 10 round 24 for 2019/20 (see Supplementary table S2).^22^ In the base case, we assumed that half of the death due to MI (i.e. fatal-MI) occurred while hospitalized and, therefore, would incur acute events cost.^6,8^ Furthermore, we assumed that the acute event costs were the same by age and sex.^22^

Chronic management costs were based on the predicted single-year age follow-up costs for the cohort with MI in the VAED. To generate cost inputs for the dynamic model, a generalized linear model was fit with a spline effects of age and time since index MI, and an interaction between the two, stratified by sex. These models were then used to estimate the chronic management cost of MI by sex, age and year of follow-up (see Supplementary Table S3). All costs in projections were adjusted to 2019 AUD using the Health Price Index.^15^

#### Estimating Transition Probabilities for the Dynamic Multistate Markov Model

Proportions and rates used in the dynamic multistate Markov model were estimated using generalized linear models. Full details of the models applied to estimate the rates and proportion have been described elsewhere.^13^ The estimated rates were converted to transition probabilities.^23^

#### Outcomes

Once the model was populated by the transition probabilities and proportions, outcomes related to health burden of MI were projected including number of MI events and years of life lived with MI.^13^ To project the acute events cost of MI over 20 years, the acute event cost described above was multiplied by the number of non-fatal MIs and half of the projected number of fatal-MIs. Likewise, the projected chronic management cost was calculated by multiplying the projected years of life lived with MI (undiscounted) with the single year age estimated chronic management cost for cohorts in the VAED. The projected total healthcare cost was calculated by combining the projected acute events cost and chronic management cost. For all projected costs, a 5% annual discounting was applied in the base case.^23^ The 5% annual discount rate reflects the Pharmaceutical Benefits Advisory Committee guideline for costs and health benefits that occur beyond the first year.^24^ Projected costs were presented by age-group and yearly projection period, stratified by sex, at a population level.

#### Sensitivity and Scenario Analysis

To derive the 95% uncertainty intervals (UI) for the projected cost burden outcomes, Monte-Carlo simulation with 1000 iterations was performed by drawing distributions appropriate for each parameter (see Supplementary Table S4). Scenario analyses were also performed including: (a) varying the discount rate (0% and 3%), (b) considering all people with fatal-IS would incur acute event costs and (c) changing the duration of projection from 20 to 10 years (2019–2028). All analyses were performed using Stata version 18.0 (StataCorp, USA).

#### Model Validation

Model validation was performed based on the assessment of the Validation Status of Health Economics decision models.^25^ To check face validity, we compared our projected, undiscounted total healthcare cost of MI for 2019 to 2021 to the AIHW reported cost of coronary heart disease (CHD) for year 2019 to 2021 (for further details, see Supplementary material page 19 and Supplementary Figure S4).^15,26,27^

## RESULT

### Current Annual Chronic Management Costs Following Myocardial Infarction

A total of 59,260 people with MI (with 135,649-person years of follow-up) were included in the study and followed for up to 6 years. The overall adjusted annual chronic management cost per person was AUD 14,412 (95% CI 14,282, 14,542). People with NSTEMI had higher chronic management costs per person, 15,112 AUD (95% CI 14,957, 15,266), compared to people diagnosed with STEMI, AUD 12,052 (95% CI 11,834, 12,270). Males incurred higher chronic management cost per person than females, AUD 14,666 (95% CI 14,499, 14,833) and AUD 13,959 (95%CI 13,748, 14,171), respectively (Table 1).

**Table 1:** Estimated Chronic Management Cost Following Myocardial Infarction Using Cohorts in the Victorian Admitted Episode Dataset

Variable	*N*	Person-years of follow-up	Total chronic cost per person (in AUD)*			Total chronic cost per person (in AUD)*		
			Unadjusted	LB	UB	Adjusted**	LB	UB
Overall	59,260	135,649	13,848	13,738	13,957	14,412	14,282	14,542
NSTEMI	44,047	97,421	15,100	14,959	15,240	15,112	14,957	15,266
STEMI	15,213	38,228	10,657	10,498	10,816	12,052	11,834	12,270
Sex								
Male	39,690	92,501	13,624	13,493	13,754	14,666	14,499	14,833
Female	19,570	43,148	14,327	14,126	14,528	13,959	13,748	14,171
Age (in years)								
30–39	983	2601	8319	7797	8839	9319	8684	9954
40–49	4602	12,124	8571	8371	8771	9101	8876	9325
50–59	10,330	27,216	9465	9308	9621	10,097	9921	10,274
60–69	13,772	34,798	13,168	12,966	13,370	13,775	13,550	14,000
70–79	13,777	31,196	17,625	17,349	17,901	17,294	17,013	17,575
≥ 80	15,796	27,714	16,511	16,275	16,745	16,870	16,608	17,132
Hypertension								
Yes	55,147	124,565	14,479	14,359	14,600	14,618	14,484	14,751
No	4113	11,083	6746	6558	6935	9676	9289	10,064
Diabetes								
Yes	18,135	37,343	19,994	19,693	20,296	18,994	18,692	19,296
No	41,125	98,305	11,513	11,406	11,620	12,289	12,163	12,416
IRSD quintile								
1 (most disadvantaged)	12,100	26,417	14,814	14,623	15,004	14,509	14,301	14,717
2	11,385	25,299	14,518	14,397	14,639	14,436	14,301	14,573
3	11,285	26,223	14,366	14,250	14,480	14,399	14,268	14,530
4	12,257	28,158	14,193	14,057	14,330	14,355	14,195	14,515
5 (least disadvantaged)	9778	22,774	14,048	13,877	14,219	14,318	14,116	14,519
Follow-up								
Year 1	59,260	47,766	15,454	15,311	15,596	15,870	15,712	16,026
Year 2	40,297	34,580	12,910	12,801	13,020	13,420	13,298	13,542
Year 3	29,268	24,657	11,085	10,962	11,210	11,677	11,537	11,817
Year 4	20,265	16,381	9713	9575	9851	10,360	10,204	10,516
Year 5	12,645	9340	8643	8498	8787	9323	9158	9488
Year 6	5956	2925	7785	7638	7932	8482	8313	8652

*N* number of people with myocardial infarction, *NSTEMI* non-ST-segment elevated myocardial infarction, *STEMI* ST-elevated myocardial infarction, *IRSD* index of relative socioeconomic disadvantage, *LB* lower bound of the confidence interval (2.5%), *UB* upper bound of the confidence interval (97.5%), *AUD* Australian dollar

^*^Total chronic management cost is the composite of hospital admission cost, medication cost and Medicare Benefits Schedule cost

^**^Total chronic management cost adjusted for age, sex, hypertension, diabetes, index of relative socioeconomic disadvantage and follow-up time

Chronic management cost per person increased with age. People in the most socioeconomically disadvantaged quintile (first quintile) had the highest chronic management cost per person. The annual chronic management cost per person declined with years of follow-up, where the highest cost per person incurred in the first year of follow-up, AUD 15,870 (95% CI 15,712, 16,026). There was a slight increase in the chronic management cost after adjusting for covariates compared to the unadjusted estimates across the sociodemographic, clinical and socioeconomic status variables.

Crude estimates of hospital admission cost, MBS cost and medication cost also exhibited the same patterns as the adjusted annual chronic management cost per person (see Supplementary Table S5 and Supplementary Figure S1-Supplemetary Figure S3). Hospital admission cost accounted the highest proportion 63.5% (AUD 8789) of the total healthcare cost followed by drug cost 19.0% (AUD 2636) and Medicare cost 17.5% (AUD 2422) (see Supplementary Table S5).

### Projection of the Cost Burden of Myocardial Infarction for the Australian Population Using a Dynamic Model

**Figure 2:**
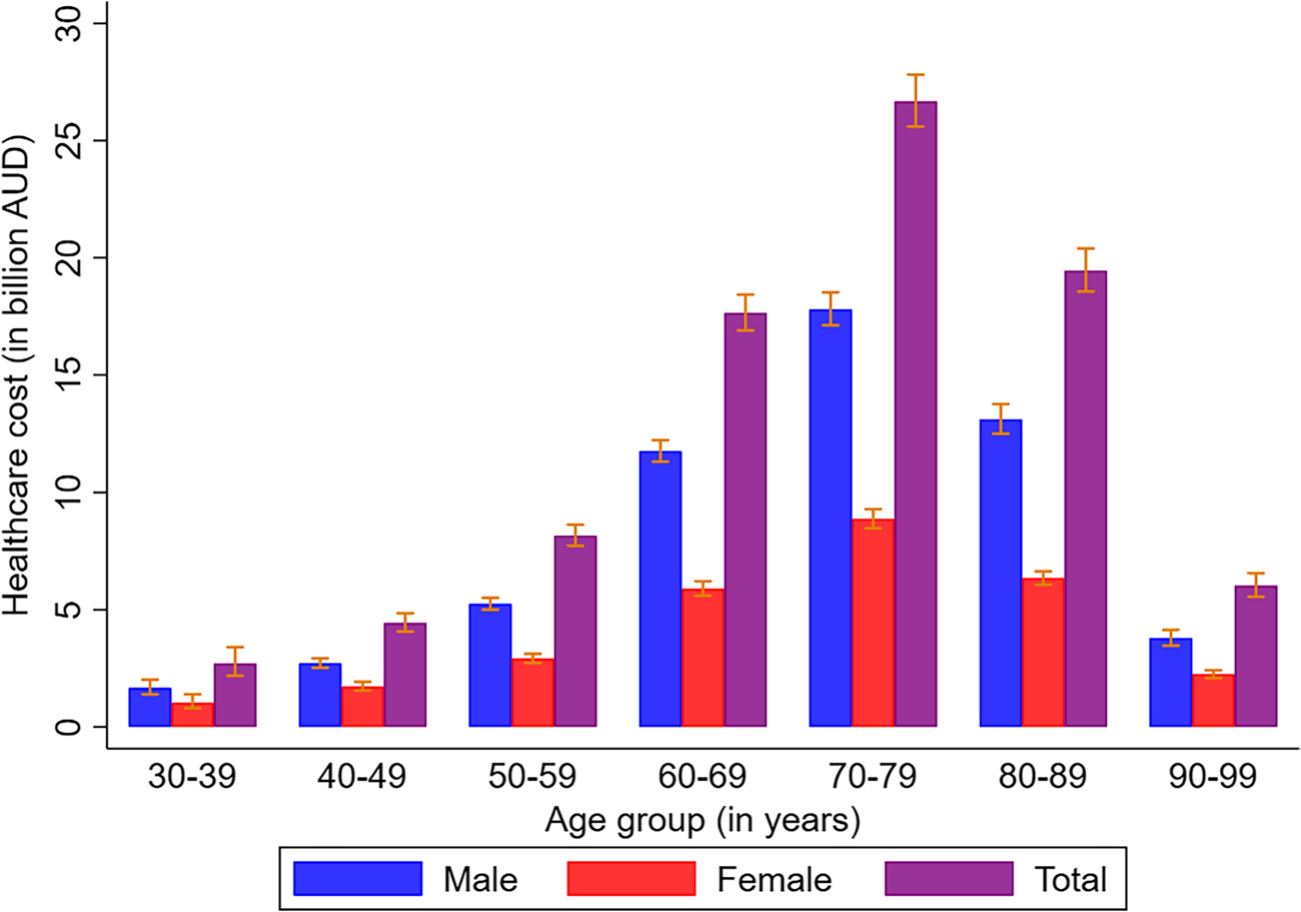
Projected healthcare cost of Myocardial infarction for Australian population aged between 30 and 99 years over 20 years (2019–2038) stratified by sex. AUD, Australian dollar.

The projected total healthcare cost of MI was AUD 85.1 billion (95% UI 80.8 billion, 89.8 billion) from 2019 to 2038, with chronic management cost accounting for 93.6% (AUD 79.6 billion) and acute events cost accounting for 6.4% (AUD 5.5 billion) of the total healthcare cost (see Fig. 2, Tables 2 and 3 and Supplementary table S6). The cost burden was higher for males than females throughout the projection period (see Supplementary table S7-S15). Stratification of the cost burden by age-group and sex also showed higher cost burden for males than females across the age groups (see Tables 2 and 3, Fig. 2 and Supplementary Table S6). The highest cost burden was observed in 70–79 years age-group in both sexes (Tables 2 and 3, Fig. 2 and Supplementary table S6).

**Table 2:** Projected Acute Events Cost of Myocardial Infarction by Age Group for the Australian Population aged 30–99 Years Over the 20-Year Period (2019–2038)

Age group	N*	Acute cost	LB	UB
Projected acute cost of myocardial infarction for male population				
30–39	34,410	214,998,330	173,460,975	260,874,800
40–49	53,857	333,075,606	271,228,822	403,363,390
50–59	80,192	502,328,696	409,161,484	605,812,936
60–69	118,738	740,540,772	602,516,116	889,044,688
70–79	148,647	907,675,976	735,770,896	1,097,754,584
80–89	123,505	720,355,908	587,836,324	866,915,348
90–99	48,849	271,169,817	220,782,216	326,226,007
**Total**	608,197	3,690,145,024	3,537,532,160	3,828,261,376
Projected acute cost of myocardial infarction for female population				
30–39	7977	49,943,567	39,595,004	61,123,676
40–49	14,953	92,344,909	74,593,543	112,724,911
50–59	26,700	167,096,358	134,831,359	202,428,434
60–69	48,240	300,008,850	244,049,620	363,202,356
70–79	74,304	450,966,180	367,700,570	546,060,436
80–89	79,267	465,254,876	380,367,176	564,013,884
90–99	43,277	250,596,123	204,136,348	302,235,794
**Total**	294,719	1,776,210,816	1,689,022,464	1,873,094,272
Projected total acute cost of myocardial infarction for total population				
30–39	42,387	264,941,897	213,055,979	321,998,476
40–49	68,810	425,420,515	345,822,365	516,088,301
50–59	106,892	669,425,054	543,992,843	808,241,370
60–69	166,978	1,040,549,622	846,565,736	1,252,247,044
70–79	222,951	1,358,642,156	1,103,471,466	1,643,815,020
80–89	202,772	1,185,610,784	968,203,500	1,430,929,232
90–99	92,126	521,765,940	424,918,564	628,461,801
**Total**	902,916	5,466,355,840	5,226,554,624	5,701,355,648

*N* number of people, *LB* lower bound of the uncertainty interval (2.5%), *UB* upper bound of the uncertainty interval (97.5%), *AUD* Australian dollar

^*^Number of people for acute events cost projection is based on the number of people with non-fatal myocardial infarction and half of the people with fatal myocardial infarction

**Table 3:** Projected Chronic Management Cost Following Myocardial Infarction by Age-Group for the Australian Population Aged 30–99 Years Over the 20-Year Period (2019–2038)

Age group	N*	Chronic cost	LB	UB
Projected chronic cost of myocardial infarction for male population				
30–39	464,606	1,451,262,144	1,176,832,720	1,796,880,848
40–49	773,028	2,383,477,344	2,205,623,088	2,584,058,864
50–59	1,204,244	4,750,677,024	4,531,126,368	4,995,023,584
60–69	1,796,451	11,003,290,624	10,593,402,304	11,453,784,128
70–79	2,180,749	16,881,441,024	16,237,848,960	17,584,345,728
80–89	1,623,738	12,380,295,296	11,802,833,152	13,008,690,432
90–99	504,584	3,511,594,456	3,203,460,600	3,855,601,792
**Total**	8,547,400	52,362,039,296	49,803,747,328	55,279,443,968
Projected chronic cost of myocardial infarction for female population				
30–39	298,082	985,087,856	746,000,472	1,339,383,208
40–49	392,488	1,626,624,760	1,460,030,560	1,831,360,160
50–59	584,356	2,744,633,280	2,561,714,352	2,951,426,608
60–69	866,801	5,594,447,200	5,307,299,712	5,899,661,344
70–79	1,069,834	8,425,865,280	8,039,659,328	8,821,699,392
80–89	888,296	5,880,180,160	5,627,954,688	6,146,802,464
90–99	379,945	1,994,671,576	1,848,151,880	2,156,077,480
**Total**	4,479,801	27,251,509,248	25,606,905,856	29,042,716,672
Projected total chronic cost of myocardial infarction for total population				
30–39	762,688	2,436,350,000	1,922,833,192	3,136,264,056
40–49	1,165,516	4,010,102,104	3,665,653,648	4,415,419,024
50–59	1,788,600	7,495,310,304	7,092,840,720	7,946,450,192
60–69	2,663,252	16,597,737,824	15,900,702,016	17,353,445,472
70–79	3,250,583	25,307,306,304	24,277,508,288	26,406,045,120
80–89	2,512,034	18,260,475,456	17,430,787,840	19,155,492,896
90–99	884,529	5,506,266,032	5,051,612,480	6,011,679,272
**Total**	13,027,201	79,613,548,544	75,410,653,184	84,322,160,640

*N* number of people, *LB* lower bound of the uncertainty interval (2.5%), *UB* upper bound of the uncertainty interval (97.5%), *AUD* Australian dollar

^*^Number of people for chronic management cost projection is based on the number of people with prevalent myocardial infarction at the beginning of each cycle

### Scenario Analyses

The projected total healthcare cost increased by 18.1% (AUD 15.4 billion) when the annual discounting rate was reduced from 5 to 3% (Table 4). Furthermore, applying no annual discounting rate, increased the projected total healthcare cost by 56.3% (AUD 47.9 billion). Assuming all fatal-MI events occurred while hospitalized increased the projected acute events cost by 1.9% (AUD 103 million) and the total healthcare cost by 0.1%. Reducing the projection period from 20 to 10 years (2019–2028) decreased the projected total healthcare cost by 41.4% (AUD 35.2 billion).

**Table 4:** Results of Scenario Analyses for Projected Acute Events, Chronic Management and Total Healthcare Cost for the Australian Population Aged 30–99 Years

Outcome	N*	Estimate (in AUD)	LB	UB
Scenario analyses for male population				
20-year projection base case analysis				
*5% discount*				
Acute cost	608,197	3,690,145,024	3,537,532,160	3,828,261,376
Chronic cost	8,547,400	52,362,039,296	49,803,747,328	55,279,443,968
Total cost**	9,155,598	56,052,183,040	53,479,501,824	58,976,018,432
***3% discount***				
Acute cost	608,197	4,387,661,824	4,278,142,976	4,626,365,952
Chronic cost	8,547,400	61,925,998,592	58,877,444,096	65,409,359,872
Total cost**	9,155,598	66,313,662,464	63,261,302,784	69,774,573,568
***0% discount***				
Acute cost	608,197	5,862,155,264	5,620,211,200	6,078,915,584
Chronic cost	8,547,400	82,078,638,080	78,004,420,608	86,698,246,144
Total cost**	9,155,598	87,940,792,320	83,872,645,120	92,534,407,168
All fatal MI^†^				
*5% discount*				
Acute cost	624,828	3,752,227,328	3,597,041,152	3,891,004,928
Chronic cost	8,547,400	52,362,039,296	49,803,747,328	55,279,443,968
Total cost	9,172,229	56,114,266,112	53,540,831,232	59,037,667,328
10-year projection				
*5% discount*				
Acute cost	268,000	2,071,633,152	1,984,139,264	2,150,857,216
Chronic cost	3,958,764	30,448,887,808	28,968,259,584	32,147,044,352
Total cost**	4,226,764	32,520,521,728	31,049,043,968	34,195,828,736
Scenario analyses for female population				
20-year projection base case analysis				
*5% discount*				
Acute cost	294,719	1,776,210,816	1,689,022,464	1,873,094,272
Chronic cost	4,479,801	27,251,509,248	25,606,905,856	29,042,716,672
Total cost**	4,774,520	29,027,721,216	27,350,888,448	30,844,469,248
***3% discount***				
Acute cost	294,719	2,163,521,280	2,010,777,344	2,229,997,568
Chronic cost	4,479,801	32,067,782,656	30,104,608,768	34,180,655,104
Total cost**	4,774,520	34,182,154,240	32,189,181,952	36,333,748,224
***0% discount***				
Acute cost	294,719	2,830,135,040	2,691,332,352	2,985,469,440
Chronic cost	4,479,801	42,176,221,184	39,571,988,480	44,982,644,736
Total cost**	4,774,520	45,006,352,384	42,387,193,856	47,859,097,600
All fatal MI^†^				
*5% discount*				
Acute cost	305,646	1,817,300,992	1,729,118,720	1,915,579,776
Chronic cost	4,479,801	27,251,509,248	25,606,905,856	29,042,716,672
Total cost	4,785,447	29,068,810,240	27,391,600,640	30,886,912,000
10-year projection				
*5% discount*				
Acute cost	128,509	989,287,744	939,698,304	1,042,637,440
Chronic cost	2,168,902	16,373,737,472	15,444,387,840	17,438,158,848
Total cost**	2,297,411	17,363,025,920	16,419,580,928	18,449,604,608
Scenario analyses for total population				
20-year projection base case analysis				
*5% discount*				
Acute cost	902,916	5,466,355,840	5,226,554,624	5,701,355,648
Chronic cost	13,027,201	79,613,548,544	75,410,653,184	84,322,160,640
Total cost**	13,930,118	85,079,904,256	80,830,390,272	89,820,487,680
***3% discount***				
Acute cost	902,916	6,551,183,104	6,288,920,320	6,856,363,520
Chronic cost	13,027,201	93,993,781,248	88,982,052,864	99,590,014,976
Total cost**	13,930,118	100,495,816,704	95,450,484,736	106,108,321,792
***0% discount***				
Acute cost	902,916	8,692,290,304	8,311,543,552	9,064,385,024
Chronic cost	13,027,201	124,254,859,264	117,576,409,088	131,680,890,880
Total cost**	13,930,118	132,947,144,704	126,259,838,976	140,393,504,768
All fatal MI^†^				
*5% discount*				
Acute cost	930,474	5,569,528,320	5,326,159,872	5,806,584,704
Chronic cost	13,027,201	79,613,548,544	75,410,653,184	84,322,160,640
Total cost	13,957,676	85,183,076,352	80,932,431,872	89,924,579,328
10-year projection				
*5% discount*				
Acute cost	396,509	3,060,920,896	2,923,837,568	3,193,494,656
Chronic cost	6,127,666	46,822,625,280	44,412,647,424	49,585,203,200
Total cost**	6,524,175	49,883,547,648	47,468,624,896	52,645,433,344

*N* number of people, *LB* lower bound of the 95% uncertainty interval (2.5%), *UB* upper bound of the 95% uncertainty interval (97.5%), *AUD* Australian dollar

^*^Number of people for acute events cost projection is based on the number of people with non-fatal myocardial infarction and half of the people with fatal myocardial infarction

Number of people for chronic management cost projection is based on the number of people with prevalent myocardial infarction at the beginning of each cycle

Number of people for total healthcare cost projection is the sum of people considered for acute events and chronic management cost projection

^**^Total cost is the sum of acute events cost and chronic management cost (i.e. total healthcare cost)

^†^Assuming that all fatal myocardial infarction events happened after hospitalization in the dynamic model and full costs is implemented

## DISCUSSION

The overall current annual chronic management cost of MI was estimated to be AUD 14,412 per person. The projection of the healthcare cost burden of MI showed an increasing burden for the Australian healthcare system for the coming years incurring a total of AUD 85.1 billion between 2019 and 2038.

Our study showed that hospital admission cost accounted for the highest proportion of the total chronic management cost per person. Consistently, Ademi et al. also reported people with coronary artery disease incurred the highest cost in hospitalization.^28^

Our study also showed a higher overall chronic management cost in the first-year follow-up and gradually declined afterwards. This was consistent with multiple studies which have demonstrated higher proportion of readmissions within the first 12 months (most importantly the first 30 days) after index MI because of cardiovascular- and non-cardiovascular reasons.^11,29^ These readmissions incur resource use ranging from investigation to treatment.^28,30^

A higher chronic management cost was observed in our study for people in most socioeconomically disadvantaged quintile. Socioeconomic disadvantage plays a crucial role in the burden of MI, with studies indicating higher hospital readmission and mortality rates among those in the most disadvantaged quintile.^6,11,31^

The cost burden of MI is expected to increase for the coming years as per our dynamic model projection. Our findings uphold a previous study conducted by Marquina et al. which projected the cost burden of CVD (mainly MI and stroke) in Australia.^8^ Differences in cost burden between our study (AUD 46.8 billion over 10 years) and Marquina et al. (AUD 61.9 billion)^8^ could be attributed to the conditions selected for the model and the types of data sources retrieved to model the cost projection.

The projected healthcare cost burden of MI indicates the significance of proactive prevention strategies to reduce the disease burden for the Australian population. Priority setting in population-wide primary prevention strategies will have a profound effect on curbing the incidence of MI.^32,33^ Secondary prevention strategies are also important in reducing the recurrence of MI and could curb follow-up costs related to rehospitalization and have been shown to be cost-effective.^34^

### Strengths and Limitations

The main strength of our study was the use of a large, representative dataset, the VAED, to estimate the current and future burden cost burden of MI. Linking multiple data sources to derive granular cost data also ensured we captured all costs for all individuals, without recall bias.

However, our study has several limitations. First, costs related to acute events were assumed to be similar regardless age and sex. Second, a conventional approach was taken in projecting the health burden of MI where rates and proportions were considered to remain stable throughout the projection time.^13^ As a result, the projected health burden of MI has likely been overestimated^13^ as studies showed that conventional approach of projecting the mortalities associated with CHD was higher than trend-based projections.^35,36^ Consequently, our projection of the healthcare cost burden of MI has also likely been overestimated. Thus, consideration should be given while interpreting the projection estimates where the results might change based on the assumed rates and proportions used for the dynamic model. Third, while the Victorian population (our data source) is likely representative of the Australian population, it is possible there are differences between Victoria and the rest of Australia.

Fourth, productivity loss due to MI was not considered in our study.^4,8^ Furthermore, costs associated with social and informal care were also not considered in our study.^7^ Hence, our estimated current and projected future cost burden of MI would have even been higher if productivity loss, social and informal care costs were also included in the model.

Lastly, we were unable to validate our model using publicly available data on the total cost of MI. However, we conducted face validation using the estimated cost of CHD provided by AIHW.^15,26,27^ Our CVD cost estimates among people with MI were slightly lower than the estimates from the AIHW’s for CVD costs attributable to CHD (for further details, see Supplementary Material Page 19 and Supplementary Figure S4). This discrepancy can likely be attributed, at least in part, to the inclusion of non-MI conditions (e.g. angina) in the AIHW’s CHD cost estimates.

## CONCLUSION

Our projections suggest that MI will cost the Australian healthcare system over AUD 85 billion in the coming years. Cost estimates based on key sociodemographic characteristics and socioeconomic disadvantage are expected to inform future health economic modelling studies for MI prevention strategies and interventions.

## Supplementary Information

Below is the link to the electronic supplementary material.

Supplementary file1 (DOCX 551 KB)

## Data Availability

The access data can be requested from the data custodians (Centre for Victorian Data Linkage and AIHW) via an application process.
