# Supplementary material for: Current and Future Cost Burden of Myocardial Infarction in Australia: Dynamic Multistate Markov Model
Source: J Gen Intern Med. 2025 Mar 4;41(2):461–70. doi: 10.1007/s11606-025-09423-8 (PMC12894549; doi:10.1007/s11606-025-09423-8)
Supplement: Supplementary file 1 — Supplementary file1 (DOCX 551 KB) [file 11606_2025_9423_MOESM1_ESM.docx]

**Supplementary materials**

**Title: Current and Future Total Cost Burden of Myocardial Infarction in Australia: Dynamic Model**

Tamrat Befekadu Abebe MMedSc^1*^, Jenni Ilomaki PhD^1,2^, Adam Livori MClinPharm^1,3^, J. Simon Bell PhD^1,2,4^, Jedidiah I Morton PhD †^1,5^ and Zanfina Ademi PhD†^1,2,4,6^

1. Centre for Medicine Use and Safety, Faculty of Pharmacy and Pharmaceutical Sciences, Monash University, Melbourne, Australia
2. School of Public Health and Preventive Medicine, Monash University, Melbourne, Australia
3. Pharmacy Department, Grampians Health Ballarat, Melbourne, Australia
4. Faculty of Health Sciences, University of Eastern Finland, Kuopio, Finland
5. Baker Heart and Diabetes Institute, Melbourne, Australia
6. Central Clinical School, Monash University, Melbourne, Australia

† Jedidiah I Morton and Zanfina Ademi share last authorship.

*****Correspondence to: Tamrat Befekadu Abebe

ORCiD ID: 0000-0002-9367-7592

Address: 407 Royal Parade, Parkville, Victoria, 3052

E-mail: [tamrat.abebe@monash.edu](mailto:tamrat.abebe@monash.edu)

**Content**

| Supplementary table | Title | Page |
| --- | --- | --- |
| Supplementary table S1 | Australia population by age and sex in 2018 | 1 |
| Supplementary table S2 | Acute events cost of Myocardial infarction | 2 |
| Supplementary table S3 | Predicted chronic management cost following Myocardial infarction for cohorts in the Victorian Admitted Episode Dataset | 6 |
| Supplementary table S4 | Input cost variables and distributions applied for the Monte-Carlo Simulation | 7 |
| Supplementary table S5 | Crude chronic management cost following Myocardial infarction for cohorts in the Victorian Admitted Episode Dataset | 8 |
| Supplementary table S6 | Projected total healthcare cost (acute events and chronic management cost) of Myocardial infarction by age-group for the Australian population aged 30-99 years over the 20-year period (2019-2038) | 9 |
| Supplementary table S7 | Projected acute events cost of Myocardial infarction by year for the male Australian population aged 30-99 years over the 20-year period (2019-2038) | 10 |
| Supplementary table S8 | Projected acute events cost of Myocardial infarction by year for the female Australian population aged 30-99 years over the 20-year period (2019-2038) | 11 |
| Supplementary table S9 | Projected acute events cost of Myocardial infarction by year for the total Australian population aged 30-99 years over the 20-year period (2019-2038) | 12 |
| Supplementary table S10 | Projected chronic management cost following Myocardial infarction by year for the male Australian population aged 30-99 years over the 20-year period (2019-2038) | 13 |
| Supplementary table S11 | Projected chronic management cost following Myocardial infarction by year for the female Australian population aged 30-99 years over the 20-year period (2019-2038) | 14 |
| Supplementary table S12 | Projected chronic management cost following Myocardial infarction by year for the total Australian population aged 30-99 years over the 20-year period (2019-2038) | 15 |
| Supplementary table S13 | Projected total healthcare cost (acute events and chronic management cost) of Myocardial infarction by year for male Australian population aged 30-99 years over the 20-year period (2019-2038) | 16 |
| Supplementary table S14 | Projected total healthcare cost (acute events and chronic management cost) of Myocardial infarction by year for female Australian population aged 30-99 years over the 20-year period (2019-2038) | 17 |
| Supplementary table S15 | Projected total healthcare cost (acute events and chronic management cost) of Myocardial infarction by year for the total Australian population aged 30-99 years over the 20-year period (2019-2038) | 18 |

| Supplementary figure | Title | Page |
| --- | --- | --- |
| Supplementary figure S1 | Average drug cost for chronic management following Myocardial infarction from Pharmaceutical Benefits Agency | 3 |
| Supplementary figure S2 | Average Medicare cost for chronic management following Myocardial infarction from Medicare Benefits Schedule | 4 |
| Supplementary figure S3 | Average admission cost for chronic management following Myocardial infarction from National Hospital Cost Data Collection. | 5 |
| Supplementary figure S4 | Model validation comparing between the projected total healthcare cost of Myocardial infarction in Australia and estimated cost of coronary heart disease from Australian Institute of Health and Welfare for year 2019-2021 | 20 |

Supplementary table S1. Australia population by age and sex in 2018

| Age | Population | |
| --- | --- | --- |
|  | Male | Female |
| 30 | 185 423 | 188 760 |
| 31 | 184 269 | 187 597 |
| 32 | 185 592 | 188 585 |
| 33 | 183 918 | 187 279 |
| 34 | 183 367 | 185 787 |
| 35 | 182 846 | 184 286 |
| 36 | 177 524 | 178 445 |
| 37 | 172 757 | 174 194 |
| 38 | 164 960 | 167 259 |
| 39 | 160 393 | 163 224 |
| 40 | 157 149 | 159 775 |
| 41 | 156 529 | 158 685 |
| 42 | 157 943 | 159 119 |
| 43 | 158 671 | 160 441 |
| 44 | 162 443 | 164 362 |
| 45 | 164 844 | 168 505 |
| 46 | 170 053 | 175 141 |
| 47 | 170 904 | 177 892 |
| 48 | 160 785 | 166 076 |
| 49 | 157 579 | 164 067 |
| 50 | 152 363 | 157 948 |
| 51 | 148 797 | 153 748 |
| 52 | 149 037 | 153 494 |
| 53 | 149 336 | 153 956 |
| 54 | 154 319 | 158 983 |
| 55 | 155 581 | 160 293 |
| 56 | 154 530 | 159 125 |
| 57 | 152 632 | 158 413 |
| 58 | 146 551 | 153 404 |
| 59 | 143 206 | 149 600 |
| 60 | 139 573 | 147 122 |
| 61 | 135 810 | 143 010 |
| 62 | 133 822 | 139 895 |
| 63 | 127 856 | 135 753 |
| 64 | 124 138 | 132 462 |
| 65 | 123 315 | 130 275 |
| 66 | 119 937 | 126 026 |
| 67 | 118 563 | 124 583 |
| 68 | 115 855 | 121 592 |
| 69 | 112 483 | 117 199 |
| 70 | 113 032 | 116 710 |
| 71 | 115 260 | 119 002 |
| 72 | 96 058 | 99 272 |
| 73 | 90 000 | 94 374 |
| 74 | 84 360 | 89 095 |
| 75 | 74 719 | 79 955 |
| 76 | 72 808 | 77 799 |
| 77 | 66 041 | 72 285 |
| 78 | 62 032 | 68 939 |
| 79 | 57 204 | 65 371 |
| 80 | 52 498 | 61 297 |
| 81 | 48 489 | 57 613 |
| 82 | 43 749 | 53 499 |
| 83 | 38 451 | 48 921 |
| 84 | 34 906 | 45 038 |
| 85 | 31 224 | 42 297 |
| 86 | 28 169 | 39 074 |
| 87 | 25 564 | 37 398 |
| 88 | 22 636 | 34 121 |
| 89 | 18 594 | 29 796 |
| 90 | 15 545 | 26 513 |
| 91 | 12 447 | 22 618 |
| 92 | 9 983 | 19 292 |
| 93 | 7 446 | 15 622 |
| 94 | 5 619 | 12 360 |
| 95 | 3 897 | 9 529 |
| 96 | 2 817 | 7 155 |
| 97 | 1 881 | 5 238 |
| 98 | 1 226 | 3 388 |
| 99 | 663 | 2 041 |

Supplementary table S2. Acute events cost of Myocardial infarction

| Acute events | Cost per event (in AUD) ^a^ | Standard error ^b^ |
| --- | --- | --- |
| Non-fatal Myocardial infarction | 9 739 | 97.39 |
| Fatal Myocardial infarction | 6 065 | 60.65 |

AUD: Australian dollar

^a^ Acute events cost was sourced from the National Hospital Cost Data Collection Version 10.0 Round 24 (2019/20) since data regarding acute events cost was not available through the Victorian Admitted Episodes Datasets.

^b^ Standard error of the acute events cost was not reported by the National Hospital Cost Data Collection. Hence 10% of the point estimate was assumed to be the standard error.


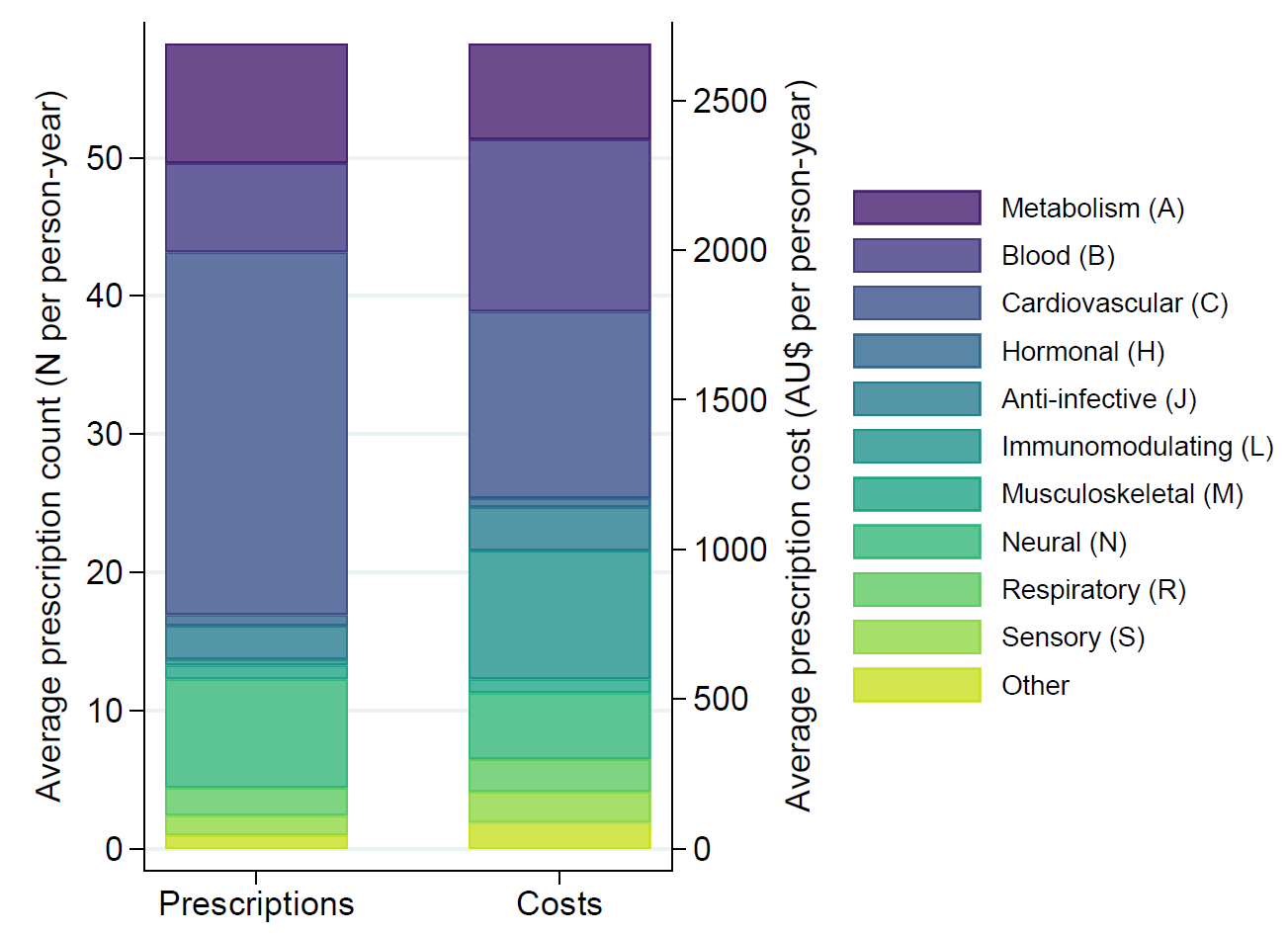


Supplementary figure S1. Average drug cost for chronic management following Myocardial infarction from Pharmaceutical Benefits Scheme (PBS)

AU$: Australian Dollar

All PBS costs were post-Myocardial infarction and could be borne by cardiovascular and non-cardiovascular conditions.


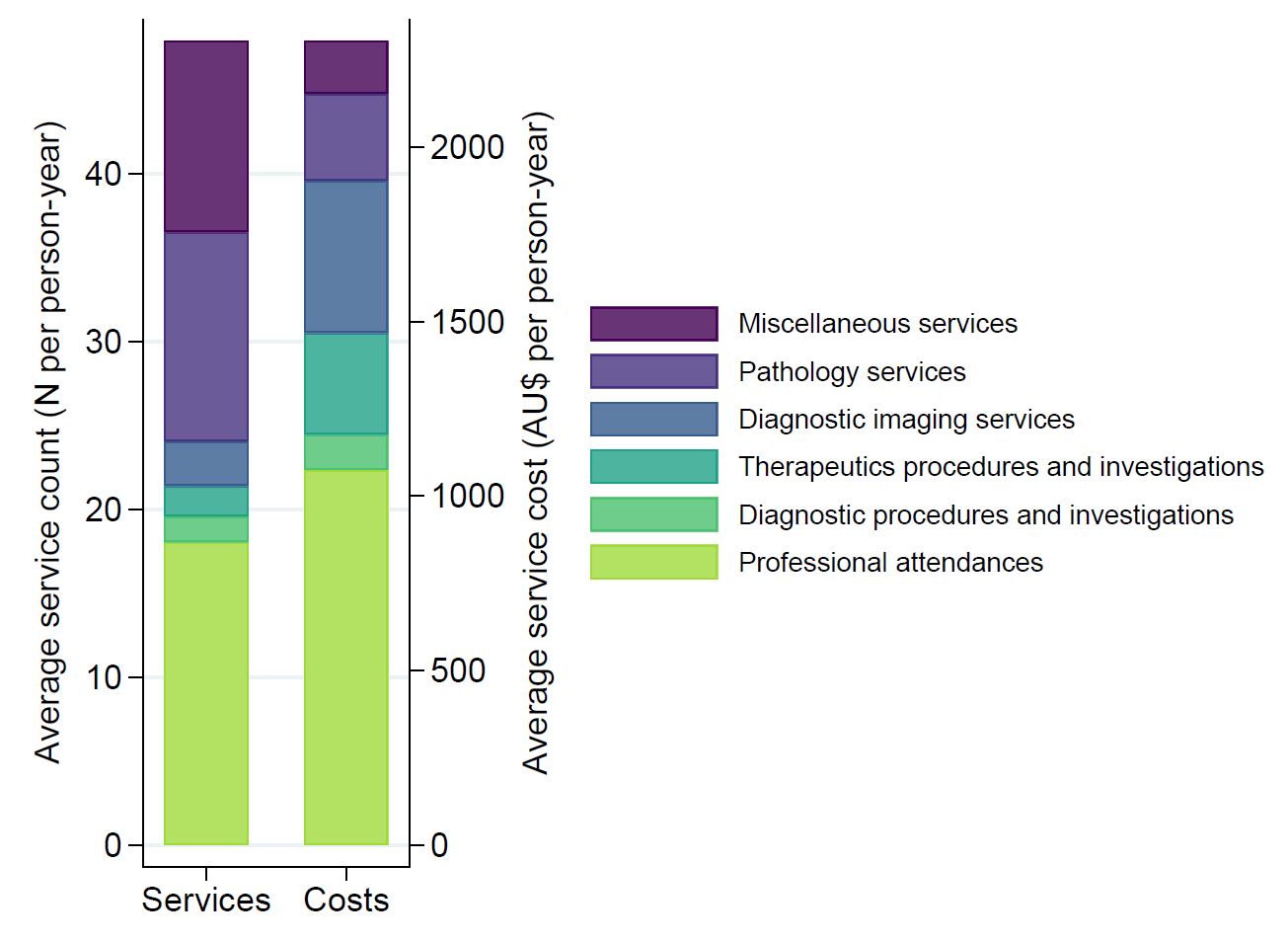


Supplementary figure S2. Average Medicare cost for chronic management following Myocardial infarction from Medicare Benefits Schedule (MBS)

AU$: Australian Dollar

All MBS costs were post-Myocardial infarction and could be borne by cardiovascular and non-cardiovascular conditions.


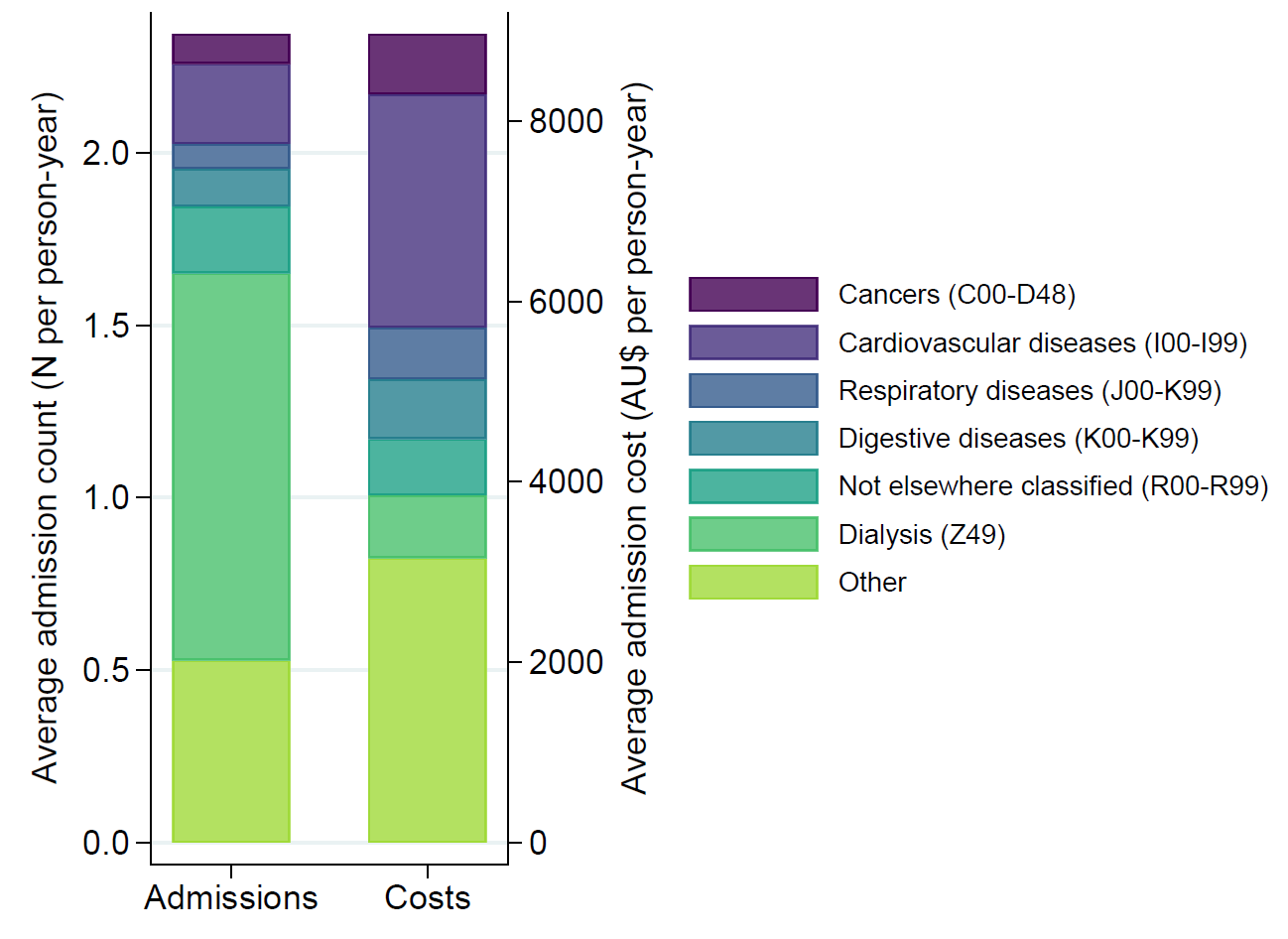


Supplementary figure S3. Average admission cost for chronic management following Myocardial infarction from National Hospital Cost Data Collection.

AU$: Australian Dollar

All admission costs were post-Myocardial infarction and could be borne by cardiovascular and non-cardiovascular conditions.

Supplementary table S3. Predicted chronic management cost following Myocardial infarction for cohorts in the Victorian Admitted Episode Dataset

| Age | Male | | Female | |
| --- | --- | --- | --- | --- |
|  | Chronic cost (in AUD) | Standard error | Chronic cost (in AUD) | Standard error |
| 30 | 4 787 | 0.14 | 4 547 | 0.21 |
| 31 | 4 778 | 0.13 | 4 646 | 0.20 |
| 32 | 4 768 | 0.12 | 4 748 | 0.18 |
| 33 | 4 758 | 0.11 | 4 852 | 0.17 |
| 34 | 4 748 | 0.11 | 4 958 | 0.16 |
| 35 | 4 739 | 0.10 | 5 066 | 0.14 |
| 36 | 4 729 | 0.09 | 5 176 | 0.13 |
| 37 | 4 719 | 0.08 | 5 289 | 0.12 |
| 38 | 4 710 | 0.07 | 5 405 | 0.11 |
| 39 | 4 700 | 0.06 | 5 523 | 0.10 |
| 40 | 4 691 | 0.06 | 5 643 | 0.08 |
| 41 | 4 682 | 0.05 | 5 766 | 0.07 |
| 42 | 4 677 | 0.04 | 5 889 | 0.07 |
| 43 | 4 678 | 0.04 | 6 010 | 0.06 |
| 44 | 4 686 | 0.03 | 6 128 | 0.05 |
| 45 | 4 704 | 0.03 | 6 241 | 0.05 |
| 46 | 4 734 | 0.03 | 6 347 | 0.05 |
| 47 | 4 779 | 0.03 | 6 443 | 0.05 |
| 48 | 4 841 | 0.03 | 6 528 | 0.05 |
| 49 | 4 923 | 0.03 | 6 599 | 0.05 |
| 50 | 5 030 | 0.03 | 6 655 | 0.05 |
| 51 | 5 161 | 0.03 | 6 697 | 0.05 |
| 52 | 5 318 | 0.03 | 6 732 | 0.04 |
| 53 | 5 500 | 0.02 | 6 767 | 0.04 |
| 54 | 5 707 | 0.02 | 6 809 | 0.03 |
| 55 | 5 939 | 0.02 | 6 867 | 0.03 |
| 56 | 6 198 | 0.02 | 6 947 | 0.03 |
| 57 | 6 481 | 0.02 | 7 058 | 0.03 |
| 58 | 6 791 | 0.02 | 7 209 | 0.03 |
| 59 | 7 125 | 0.02 | 7 410 | 0.03 |
| 60 | 7 484 | 0.02 | 7 673 | 0.03 |
| 61 | 7 865 | 0.02 | 8 003 | 0.03 |
| 62 | 8 267 | 0.02 | 8 391 | 0.03 |
| 63 | 8 684 | 0.02 | 8 832 | 0.03 |
| 64 | 9 113 | 0.02 | 9 316 | 0.03 |
| 65 | 9 547 | 0.02 | 9 830 | 0.02 |
| 66 | 9 979 | 0.02 | 10 359 | 0.02 |
| 67 | 10 401 | 0.02 | 10 884 | 0.02 |
| 68 | 10 805 | 0.02 | 11 382 | 0.02 |
| 69 | 11 181 | 0.02 | 11 829 | 0.02 |
| 70 | 11 518 | 0.02 | 12 195 | 0.02 |
| 71 | 11 812 | 0.02 | 12 468 | 0.02 |
| 72 | 12 063 | 0.02 | 12 651 | 0.02 |
| 73 | 12 277 | 0.02 | 12 752 | 0.02 |
| 74 | 12 455 | 0.02 | 12 779 | 0.02 |
| 75 | 12 602 | 0.02 | 12 745 | 0.02 |
| 76 | 12 724 | 0.02 | 12 660 | 0.02 |
| 77 | 12 825 | 0.02 | 12 537 | 0.02 |
| 78 | 12 912 | 0.02 | 12 387 | 0.02 |
| 79 | 12 990 | 0.02 | 12 223 | 0.02 |
| 80 | 13 065 | 0.02 | 12 056 | 0.02 |
| 81 | 13 141 | 0.02 | 11 891 | 0.02 |
| 82 | 13 217 | 0.02 | 11 728 | 0.02 |
| 83 | 13 293 | 0.02 | 11 567 | 0.02 |
| 84 | 13 370 | 0.02 | 11 408 | 0.02 |
| 85 | 13 448 | 0.02 | 11 252 | 0.02 |
| 86 | 13 525 | 0.02 | 11 098 | 0.02 |
| 87 | 13 604 | 0.02 | 10 945 | 0.02 |
| 88 | 13 682 | 0.03 | 10 795 | 0.02 |
| 89 | 13 761 | 0.03 | 10 647 | 0.02 |
| 90 | 13 841 | 0.03 | 10 501 | 0.02 |
| 91 | 13 921 | 0.03 | 10 357 | 0.03 |
| 92 | 14 002 | 0.04 | 10 215 | 0.03 |
| 93 | 14 082 | 0.04 | 10 075 | 0.03 |
| 94 | 14 164 | 0.04 | 9 937 | 0.03 |
| 95 | 14 246 | 0.04 | 9 801 | 0.03 |
| 96 | 14 328 | 0.04 | 9 666 | 0.04 |
| 97 | 14 411 | 0.05 | 9 534 | 0.04 |
| 98 | 14 494 | 0.05 | 9 403 | 0.04 |
| 99 | 14 578 | 0.05 | 9 274 | 0.04 |

AUD: Australian dollar

Supplementary table S4. Input cost variables and distributions applied for the Monte-Carlo Simulation

| Input | Distribution | Formula applied |
| --- | --- | --- |
| Acute events cost | Gamma | gamma(alpha, beta) ^b^ |
| Predicted chronic cost of MI ^a^ | Lognormal | exp(ln(rate)+(rnorm*SE)) ^c^ |

exp: exponential; MI: Myocardial infarction; ln: natural logarithm; rnorm: standard random normal distribution; SE: Standard error

^a^ Predicted single-year age chronic management cost values for year 3 follow-up of people with Myocardial infarction in the Victorian linked dataset.

^b^ Shape (alpha) and scale (beta) parameters of the acute events cost of Myocardial infarction were applied to draw the values from gamma distribution using the method of moments approach. Standard error of the acute events cost was not reported by the National Hospital Cost Data Collection. Hence, 10% of the point estimate was assumed to be the standard error.

^c^ The natural logarithm of the predicted incidence or mortality rates summed with the product of the standard errors of the corresponding predicted incidence or mortality rates and values drew from the standard random normal distribution and finally exponentiating the calculated estimates.

Supplementary table S5. Crude chronic management cost following Myocardial infarction for cohorts in the Victorian Admitted Episode Dataset

|  |  |  | Mean crude chronic management cost per person (in AUD) | | | |
| --- | --- | --- | --- | --- | --- | --- |
| Variable | N | Person-years | Admission cost (in AUD) | Drug cost  (in AUD) | Medicare cost (in AUD) | Total cost  (in AUD) |
| Overall | 59 260 | 135 649 | 8 789 | 2 636 | 2 422 | 13 847 |
| NSTEMI | 44 047 | 97 421 | 9 777 | 2 768 | 2 555 | 15 099 |
| STEMI | 15 213 | 38 228 | 6 271 | 2 303 | 2 082 | 10 657 |
| Sex |  |  |  |  |  |  |
| Male | 39 690 | 92 501 | 8 668 | 2 623 | 2 333 | 13 624 |
| Female | 19 570 | 43 148 | 9 049 | 2 668 | 2 611 | 14 327 |
| Age (in years) |  |  |  |  |  |  |
| 30-39 | 983 | 2 601 | 4 975 | 1 835 | 1 408 | 8 219 |
| 40-49 | 4 602 | 12 124 | 4 412 | 1 905 | 1 493 | 7 811 |
| 50-59 | 10 330 | 27 216 | 5 938 | 2 210 | 1 802 | 9 949 |
| 60-69 | 13 772 | 34 798 | 8 457 | 2 667 | 2 362 | 13 486 |
| 70-79 | 13 777 | 31 196 | 11 569 | 3 107 | 3 148 | 17 823 |
| ≥80 | 15 796 | 27 714 | 11 150 | 2 885 | 2 789 | 16 825 |
| Hypertension |  |  |  |  |  |  |
| Yes | 55 147 | 124 565 | 9 201 | 2 757 | 2 522 | 14 479 |
| No | 4 113 | 11 083 | 4 165 | 1 280 | 1 301 | 6 746 |
| Diabetes |  |  |  |  |  |  |
| Yes | 18 135 | 37 343 | 13 326 | 3 647 | 3 021 | 19 994 |
| No | 41 125 | 98 305 | 7 066 | 2 253 | 2 194 | 11 513 |
| IRSD quintile |  |  |  |  |  |  |
| 1 | 12 100 | 26 417 | 9 791 | 2 911 | 2 433 | 15 136 |
| 2 | 11 385 | 25 299 | 8 617 | 2 709 | 2 386 | 13 712 |
| 3 | 11 285 | 26 223 | 8 991 | 2 746 | 2 522 | 14 259 |
| 4 | 12 257 | 28 158 | 9 043 | 2 766 | 2 674 | 14 482 |
| 5 | 9 778 | 22 774 | 9 008 | 2 708 | 2 723 | 14 438 |
| Follow-up |  |  |  |  |  |  |
| Year 1 | 59 260 | 47 766 | 12 028 | 3 135 | 2 976 | 18 139 |
| Year 2 | 40 297 | 34 580 | 7 775 | 2 546 | 2 201 | 12 523 |
| Year 3 | 29 268 | 24 657 | 6 857 | 2 363 | 2 104 | 11 324 |
| Year 4 | 20 265 | 16 381 | 6 378 | 2 191 | 2 060 | 10 629 |
| Year 5 | 12 645 | 9 340 | 6 224 | 2 124 | 2 000 | 10 347 |
| Year 6 | 5 956 | 2 925 | 5 861 | 2 018 | 2 017 | 9 895 |

AUD: Australian dollar; N: number of people with Myocardial infarction; NSTEMI: Non-ST-Segment Elevated Myocardial Infarction; STEMI: ST-Elevated Myocardial Infarction; IRSD: Index of Relative Socioeconomic Disadvantage (first quintile: most disadvantaged; fifth quintile: least disadvantaged).

Supplementary table S6. Projected total healthcare cost (acute events and chronic management cost) of Myocardial infarction by age-group for the Australian population aged 30-99 years over the 20-year period (2019-2038)

| Projected total healthcare cost of Myocardial infarction for male population | | | | |
| --- | --- | --- | --- | --- |
| Age group | N* | Healthcare cost | LB | UB |
| 30-39 | 499 016 | 1 666 260 480 | 1 387 390 624 | 2 015 056 208 |
| 40-49 | 826 885 | 2 716 552 928 | 2 521 173 424 | 2 925 173 504 |
| 50-59 | 1 284 436 | 5 253 005 696 | 5 003 801 216 | 5 510 623 680 |
| 60-69 | 1 915 189 | 11 743 831 296 | 11 298 281 856 | 12 213 681 536 |
| 70-79 | 2 329 396 | 17 789 116 800 | 17 119 304 320 | 18 520 057 472 |
| 80-89 | 1 747 243 | 13 100 651 200 | 12 492 186 560 | 13 755 171 968 |
| 90-99 | 553 433 | 3 782 764 232 | 3 469 070 400 | 4 138 614 192 |
| Total | 9 155 598 | 56 052 183 040 | 53 479 501 824 | 58 976 018 432 |
| Projected total healthcare cost of Myocardial infarction for female population | | | | |
| Age group | N* | Healthcare cost | LB | UB |
| 30-39 | 306 058 | 1 035 031 432 | 797 196 896 | 1 390 598 352 |
| 40-49 | 407 441 | 1 718 969 696 | 1 549 786 688 | 1 924 504 432 |
| 50-59 | 611 056 | 2 911 729 680 | 2 724 066 976 | 3 118 093 440 |
| 60-69 | 915 041 | 5 894 456 000 | 5 598 750 912 | 6 211 087 648 |
| 70-79 | 1 144 139 | 8 876 831 488 | 8 475 248 640 | 9 290 724 288 |
| 80-89 | 967 563 | 6 345 435 040 | 6 066 827 424 | 6 637 814 688 |
| 90-99 | 423 222 | 2 245 267 744 | 2 083 705 696 | 2 415 790 720 |
| Total | 4 774 520 | 29 027 721 216 | 27 350 888 448 | 30 844 469 248 |
| Projected total healthcare cost of Myocardial infarction for the total population | | | | |
| Age group | N* | Healthcare cost | LB | UB |
| 30-39 | 805 074 | 2 701 291 912 | 2 184 587 520 | 3 405 654 560 |
| 40-49 | 1 234 326 | 4 435 522 624 | 4 070 960 112 | 4 849 677 936 |
| 50-59 | 1 895 492 | 8 164 735 376 | 7 727 868 192 | 8 628 717 120 |
| 60-69 | 2 830 230 | 17 638 287 296 | 16 897 032 768 | 18 424 769 184 |
| 70-79 | 3 473 535 | 26 665 948 288 | 25 594 552 960 | 27 810 781 760 |
| 80-89 | 2 714 806 | 19 446 086 240 | 18 559 013 984 | 20 392 986 656 |
| 90-99 | 976 655 | 6 028 031 976 | 5 552 776 096 | 6 554 404 912 |
| Total | 13 930 118 | 85 079 904 256 | 80 830 390 272 | 89 820 487 680 |

AUD: Australian dollar; N: Number of people; LB: Lower bound of the uncertainty interval (2.5%); UB: Upper bound of the uncertainty interval (97.5%)

*Number of people for total healthcare cost projection is the sum of people considered for acute events cost and chronic management cost projection

Supplementary table S7. Projected acute events cost of Myocardial infarction by year for the male Australian population aged 30-99 years over the 20-year period (2019-2038)

| Projected acute events cost of Myocardial infarction for male population | | | | |
| --- | --- | --- | --- | --- |
| Year | N* | Acute cost (in AUD) | LB | UB |
| 2019 | 23 231 | 224 291 552 | 214 710 512 | 233 143 840 |
| 2020 | 24 043 | 221 032 064 | 211 685 088 | 229 704 352 |
| 2021 | 24 851 | 217 533 680 | 208 362 528 | 225 986 832 |
| 2022 | 25 651 | 213 806 608 | 204 753 952 | 222 064 240 |
| 2023 | 26 446 | 209 892 848 | 200 943 392 | 217 951 680 |
| 2024 | 27 231 | 205 799 264 | 197 130 192 | 213 654 976 |
| 2025 | 28 008 | 201 553 712 | 193 102 656 | 209 250 656 |
| 2026 | 28 768 | 197 137 488 | 188 945 120 | 204 564 352 |
| 2027 | 29 517 | 192 605 648 | 184 613 424 | 199 851 136 |
| 2028 | 30 253 | 187 980 272 | 180 173 728 | 195 005 264 |
| 2029 | 30 978 | 183 292 256 | 175 822 848 | 190 077 952 |
| 2030 | 31 686 | 178 534 016 | 171 169 520 | 185 148 480 |
| 2031 | 32 384 | 173 755 232 | 166 605 872 | 180 171 904 |
| 2032 | 33 071 | 168 967 648 | 162 101 920 | 175 137 504 |
| 2033 | 33 744 | 164 179 968 | 157 529 760 | 170 237 184 |
| 2034 | 34 405 | 159 401 936 | 152 971 744 | 165 209 984 |
| 2035 | 35 051 | 154 647 728 | 148 431 408 | 160 225 568 |
| 2036 | 35 681 | 149 913 904 | 143 850 048 | 155 342 528 |
| 2037 | 36 297 | 145 226 416 | 139 359 872 | 150 403 360 |
| 2038 | 36 899 | 140 592 880 | 134 947 936 | 145 601 856 |
| **Total** | 608 197 | 3 690 145 024 | 3 537 532 160 | 3 828 261 376 |

AUD: Australian dollar; N: Number of people; LB: Lower bound of the uncertainty interval (2.5%); UB: Upper bound of the uncertainty interval (97.5%)

*Number of people for acute events cost projection is based on the number of people with non-fatal Myocardial infarction and half of the people with fatal Myocardial infarction.

Supplementary table S8. Projected acute events cost of Myocardial infarction by year for the female Australian population aged 30-99 years over the 20-year period (2019-2038)

| Projected acute events cost of Myocardial infarction for female population | | | | |
| --- | --- | --- | --- | --- |
| Year | N* | Acute cost (in AUD) | LB | UB |
| 2019 | 11 120 | 106 911 000 | 101 368 912 | 112 735 424 |
| 2020 | 11 516 | 105 429 096 | 99 985 656 | 111 092 192 |
| 2021 | 11 905 | 103 786 864 | 98 535 776 | 109 339 312 |
| 2022 | 12 291 | 102 032 688 | 96 865 200 | 107 481 824 |
| 2023 | 12 673 | 100 182 192 | 95 145 792 | 105 523 120 |
| 2024 | 13 052 | 98 253 352 | 93 401 392 | 103 566 960 |
| 2025 | 13 428 | 96 261 016 | 91 542 296 | 101 524 808 |
| 2026 | 13 801 | 94 215 560 | 89 581 312 | 99 414 984 |
| 2027 | 14 175 | 92 150 576 | 87 623 552 | 97 195 568 |
| 2028 | 14 548 | 90 065 408 | 85 640 320 | 95 011 152 |
| 2029 | 14 924 | 87 983 176 | 83 656 216 | 92 872 256 |
| 2030 | 15 298 | 85 890 336 | 81 658 768 | 90 642 336 |
| 2031 | 15 675 | 83 807 664 | 79 696 976 | 88 480 624 |
| 2032 | 16 058 | 81 753 520 | 77 723 200 | 86 315 808 |
| 2033 | 16 439 | 79 701 520 | 75 769 472 | 84 132 856 |
| 2034 | 16 820 | 77 657 288 | 73 835 384 | 81 959 400 |
| 2035 | 17 197 | 75 608 648 | 71 919 968 | 79 785 968 |
| 2036 | 17 569 | 73 554 888 | 69 967 136 | 77 606 832 |
| 2037 | 17 935 | 71 504 376 | 68 024 880 | 75 426 864 |
| 2038 | 18 295 | 69 461 672 | 66 062 252 | 73 301 744 |
| **Total** | 294 719 | 1 776 210 816 | 1 689 022 464 | 1 873 094 272 |

AUD: Australian dollar; N: Number of people; LB: Lower bound of the uncertainty interval (2.5%); UB: Upper bound of the uncertainty interval (97.5%)

*Number of people for acute events cost projection is based on the number of people with non-fatal Myocardial infarction and half of the people with fatal Myocardial infarction.

Supplementary table S9. Projected acute events cost of Myocardial infarction by year for the total Australian population aged 30-99 years over the 20-year period (2019-2038)

| Projected total acute cost of Myocardial infarction | | | | |
| --- | --- | --- | --- | --- |
| Year | N* | Acute cost (in AUD) | LB | UB |
| 2019 | 34 351 | 331 202 552 | 316 079 424 | 345 879 264 |
| 2020 | 35 559 | 326 461 160 | 311 670 744 | 340 796 544 |
| 2021 | 36 756 | 321 320 544 | 306 898 304 | 335 326 144 |
| 2022 | 37 942 | 315 839 296 | 301 619 152 | 329 546 064 |
| 2023 | 39 119 | 310 075 040 | 296 089 184 | 323 474 800 |
| 2024 | 40 283 | 304 052 616 | 290 531 584 | 317 221 936 |
| 2025 | 41 436 | 297 814 728 | 284 644 952 | 310 775 464 |
| 2026 | 42 569 | 291 353 048 | 278 526 432 | 303 979 336 |
| 2027 | 43 692 | 284 756 224 | 272 236 976 | 297 046 704 |
| 2028 | 44 801 | 278 045 680 | 265 814 048 | 290 016 416 |
| 2029 | 45 902 | 271 275 432 | 259 479 064 | 282 950 208 |
| 2030 | 46 984 | 264 424 352 | 252 828 288 | 275 790 816 |
| 2031 | 48 059 | 257 562 896 | 246 302 848 | 268 652 528 |
| 2032 | 49 129 | 250 721 168 | 239 825 120 | 261 453 312 |
| 2033 | 50 183 | 243 881 488 | 233 299 232 | 254 370 040 |
| 2034 | 51 225 | 237 059 224 | 226 807 128 | 247 169 384 |
| 2035 | 52 248 | 230 256 376 | 220 351 376 | 240 011 536 |
| 2036 | 53 250 | 223 468 792 | 213 817 184 | 232 949 360 |
| 2037 | 54 232 | 216 730 792 | 207 384 752 | 225 830 224 |
| 2038 | 55 194 | 210 054 552 | 201 010 188 | 218 903 600 |
| **Total** | 902 916 | 5 466 355 840 | 5 226 554 624 | 5 701 355 648 |

AUD: Australian dollar; N: Number of people; LB: Lower bound of the uncertainty interval (2.5%); UB: Upper bound of the uncertainty interval (97.5%)

*Number of people for acute events cost projection is based on the number of people with non-fatal Myocardial infarction and half of the people with fatal Myocardial infarction.

Supplementary table S10. Projected chronic management cost following Myocardial infarction by year for the male Australian population aged 30-99 years over the 20-year period (2019-2038)

| Projected chronic management cost following Myocardial infarction for male population | | | | |
| --- | --- | --- | --- | --- |
| Year | N | Chronic cost (in AUD) | LB | UB |
| 2019 | 373 299 | 3 545 213 440 | 3 379 222 016 | 3 726 299 136 |
| 2020 | 377 258 | 3 414 469 888 | 3 248 879 616 | 3 590 569 472 |
| 2021 | 381 697 | 3 292 992 000 | 3 135 150 592 | 3 466 669 568 |
| 2022 | 386 553 | 3 179 520 768 | 3 025 439 232 | 3 352 074 240 |
| 2023 | 391 817 | 3 072 965 632 | 2 923 234 304 | 3 244 824 064 |
| 2024 | 397 423 | 2 972 320 256 | 2 827 788 288 | 3 136 570 880 |
| 2025 | 403 335 | 2 876 680 704 | 2 737 103 872 | 3 036 193 280 |
| 2026 | 409 440 | 2 785 195 776 | 2 649 608 448 | 2 940 056 832 |
| 2027 | 415 743 | 2 697 217 024 | 2 564 359 680 | 2 848 261 120 |
| 2028 | 422 199 | 2 612 312 576 | 2 482 699 264 | 2 758 343 168 |
| 2029 | 428 784 | 2 530 059 520 | 2 403 657 472 | 2 672 262 656 |
| 2030 | 435 423 | 2 450 048 768 | 2 326 102 016 | 2 588 763 392 |
| 2031 | 442 126 | 2 372 036 352 | 2 251 542 016 | 2 506 530 816 |
| 2032 | 448 853 | 2 295 911 424 | 2 178 511 360 | 2 426 332 672 |
| 2033 | 455 579 | 2 221 475 328 | 2 106 830 208 | 2 348 338 176 |
| 2034 | 462 291 | 2 148 589 056 | 2 036 842 752 | 2 272 072 192 |
| 2035 | 468 975 | 2 077 198 848 | 1 968 252 672 | 2 197 291 008 |
| 2036 | 475 611 | 2 007 249 152 | 1 900 947 456 | 2 123 945 728 |
| 2037 | 482 220 | 1 938 762 496 | 1 835 133 312 | 2 052 099 456 |
| 2038 | 488 777 | 1 871 819 008 | 1 770 938 496 | 1 981 997 824 |
| **Total** | 8 547 400 | 52 362 039 296 | 49 803 747 328 | 55 279 443 968 |

AUD: Australian dollar; N: Number of people; LB: Lower bound of the uncertainty interval (2.5%); UB: Upper bound of the uncertainty interval (97.5%)

*Number of people for chronic management cost projection is based on the number of people with prevalent Myocardial infarction at the beginning of each cycle

Supplementary table S11. Projected chronic management cost following Myocardial infarction by year for the female Australian population aged 30-99 years over the 20-year period (2019-2038)

| Projected chronic management cost following Myocardial infarction for female population | | | | |
| --- | --- | --- | --- | --- |
| Year | N | Chronic cost (in AUD) | LB | UB |
| 2019 | 214 635 | 1 979 201 024 | 1 867 554 304 | 2 110 806 016 |
| 2020 | 214 623 | 1 890 068 096 | 1 784 224 512 | 2 014 820 480 |
| 2021 | 214 806 | 1 806 723 328 | 1 705 367 296 | 1 924 980 480 |
| 2022 | 215 193 | 1 728 618 368 | 1 631 046 144 | 1 841 455 360 |
| 2023 | 215 782 | 1 655 304 064 | 1 561 358 848 | 1 762 779 904 |
| 2024 | 216 567 | 1 586 320 128 | 1 495 492 096 | 1 689 896 704 |
| 2025 | 217 529 | 1 521 236 224 | 1 432 324 096 | 1 621 347 840 |
| 2026 | 218 622 | 1 459 602 432 | 1 371 615 488 | 1 555 849 472 |
| 2027 | 219 880 | 1 401 123 968 | 1 315 124 480 | 1 493 947 904 |
| 2028 | 221 266 | 1 345 539 840 | 1 262 582 784 | 1 435 129 344 |
| 2029 | 222 789 | 1 292 576 512 | 1 212 649 984 | 1 378 757 888 |
| 2030 | 224 415 | 1 241 989 760 | 1 164 871 552 | 1 325 454 848 |
| 2031 | 226 140 | 1 193 553 408 | 1 119 141 120 | 1 274 188 928 |
| 2032 | 227 973 | 1 147 150 720 | 1 075 595 264 | 1 225 353 728 |
| 2033 | 229 873 | 1 102 599 808 | 1 033 997 568 | 1 178 314 240 |
| 2034 | 231 839 | 1 059 725 696 | 993 204 224 | 1 133 214 208 |
| 2035 | 233 845 | 1 018 411 264 | 953 700 480 | 1 089 858 944 |
| 2036 | 235 900 | 978 561 536 | 915 769 024 | 1 047 970 176 |
| 2037 | 238 000 | 940 133 440 | 879 393 920 | 1 007 518 464 |
| 2038 | 240 125 | 903 070 592 | 844 643 072 | 968 186 176 |
| **Total** | 4 479 801 | 27 251 509 248 | 25 606 905 856 | 29 042 716 672 |

AUD: Australian dollar; N: Number of people; LB: Lower bound of the uncertainty interval (2.5%); UB: Upper bound of the uncertainty interval (97.5%)

*Number of people for chronic management cost projection is based on the number of people with prevalent Myocardial infarction at the beginning of each cycle

Supplementary table S12. Projected chronic management cost following Myocardial infarction by year for the total Australian population aged 30-99 years over the 20-year period (2019-2038)

| Projected total chronic management cost following Myocardial infarction | | | | |
| --- | --- | --- | --- | --- |
| Year | N | Chronic cost (in AUD) | LB | UB |
| 2019 | 587 934 | 5 524 414 464 | 5 246 776 320 | 5 837 105 152 |
| 2020 | 591 881 | 5 304 537 984 | 5 033 104 128 | 5 605 389 952 |
| 2021 | 596 503 | 5 099 715 328 | 4 840 517 888 | 5 391 650 048 |
| 2022 | 601 746 | 4 908 139 136 | 4 656 485 376 | 5 193 529 600 |
| 2023 | 607 599 | 4 728 269 696 | 4 484 593 152 | 5 007 603 968 |
| 2024 | 613 990 | 4 558 640 384 | 4 323 280 384 | 4 826 467 584 |
| 2025 | 620 864 | 4 397 916 928 | 4 169 427 968 | 4 657 541 120 |
| 2026 | 628 062 | 4 244 798 208 | 4 021 223 936 | 4 495 906 304 |
| 2027 | 635 623 | 4 098 340 992 | 3 879 484 160 | 4 342 209 024 |
| 2028 | 643 465 | 3 957 852 416 | 3 745 282 048 | 4 193 472 512 |
| 2029 | 651 573 | 3 822 636 032 | 3 616 307 456 | 4 051 020 544 |
| 2030 | 659 838 | 3 692 038 528 | 3 490 973 568 | 3 914 218 240 |
| 2031 | 668 266 | 3 565 589 760 | 3 370 683 136 | 3 780 719 744 |
| 2032 | 676 826 | 3 443 062 144 | 3 254 106 624 | 3 651 686 400 |
| 2033 | 685 452 | 3 324 075 136 | 3 140 827 776 | 3 526 652 416 |
| 2034 | 694 130 | 3 208 314 752 | 3 030 046 976 | 3 405 286 400 |
| 2035 | 702 820 | 3 095 610 112 | 2 921 953 152 | 3 287 149 952 |
| 2036 | 711 511 | 2 985 810 688 | 2 816 716 480 | 3 171 915 904 |
| 2037 | 720 220 | 2 878 895 936 | 2 714 527 232 | 3 059 617 920 |
| 2038 | 728 902 | 2 774 889 600 | 2 615 581 568 | 2 950 184 000 |
| **Total** | 13 027 201 | 79 613 548 544 | 75 410 653 184 | 84 322 160 640 |

AUD: Australian dollar; N: Number of people; LB: Lower bound of the uncertainty interval (2.5%); UB: Upper bound of the uncertainty interval (97.5%)

*Number of people for chronic management cost projection is based on the number of people with prevalent Myocardial infarction at the beginning of each cycle

Supplementary table S13. Projected total healthcare cost (acute events and chronic management cost) of Myocardial infarction by year for male Australian population aged 30-99 years over the 20-year period (2019-2038)

| Projected total healthcare cost of Myocardial infarction for male population | | | | |
| --- | --- | --- | --- | --- |
| Year | N | Healthcare cost (in AUD) | LB | UB |
| 2019 | 396 530 | 3 769 505 024 | 3 600 775 680 | 3 950 272 512 |
| 2020 | 401 301 | 3 635 501 824 | 3 471 943 936 | 3 814 022 656 |
| 2021 | 406 548 | 3 510 525 696 | 3 353 245 696 | 3 688 159 488 |
| 2022 | 412 205 | 3 393 327 360 | 3 239 832 576 | 3 569 201 152 |
| 2023 | 418 262 | 3 282 858 496 | 3 134 418 688 | 3 451 644 928 |
| 2024 | 424 654 | 3 178 119 424 | 3 034 872 320 | 3 341 162 496 |
| 2025 | 431 343 | 3 078 234 368 | 2 938 619 904 | 3 237 137 920 |
| 2026 | 438 209 | 2 982 333 440 | 2 845 500 672 | 3 137 334 272 |
| 2027 | 445 260 | 2 889 822 720 | 2 756 549 888 | 3 040 043 520 |
| 2028 | 452 452 | 2 800 292 864 | 2 670 963 200 | 2 946 333 184 |
| 2029 | 459 761 | 2 713 351 680 | 2 586 404 864 | 2 855 461 376 |
| 2030 | 467 109 | 2 628 582 656 | 2 504 057 856 | 2 767 435 008 |
| 2031 | 474 510 | 2 545 791 488 | 2 424 889 600 | 2 681 276 160 |
| 2032 | 481 923 | 2 464 879 104 | 2 346 898 688 | 2 596 826 112 |
| 2033 | 489 324 | 2 385 655 296 | 2 270 515 200 | 2 514 005 504 |
| 2034 | 496 695 | 2 307 990 784 | 2 195 390 976 | 2 433 057 024 |
| 2035 | 504 026 | 2 231 846 656 | 2 122 163 200 | 2 353 579 776 |
| 2036 | 511 292 | 2 157 163 008 | 2 050 403 840 | 2 276 049 664 |
| 2037 | 518 517 | 2 083 988 992 | 1 979 913 472 | 2 199 352 320 |
| 2038 | 525 676 | 2 012 411 904 | 1 910 651 136 | 2 124 198 400 |
| **Total** | 9 155 598 | 56 052 183 040 | 53 479 501 824 | 58 976 018 432 |

AUD: Australian dollar; N: Number of people; LB: Lower bound of the uncertainty interval (2.5%); UB: Upper bound of the uncertainty interval (97.5%)

*Number of people for total healthcare cost projection is the sum of people considered for acute events cost and chronic management cost projection

Supplementary table S14. Projected total healthcare cost (acute events and chronic management cost) of Myocardial infarction by year for female Australian population aged 30-99 years over the 20-year period (2019-2038)

| Projected total healthcare cost of Myocardial infarction for female population | | | | |
| --- | --- | --- | --- | --- |
| Year | N | Healthcare cost (in AUD) | LB | UB |
| 2019 | 225 755 | 2 086 112 000 | 1 974 672 128 | 2 217 489 920 |
| 2020 | 226 139 | 1 995 497 088 | 1 888 959 744 | 2 120 145 920 |
| 2021 | 226 711 | 1 910 510 208 | 1 807 821 824 | 2 028 281 600 |
| 2022 | 227 484 | 1 830 651 136 | 1 731 510 528 | 1 945 286 144 |
| 2023 | 228 455 | 1 755 486 336 | 1 660 036 096 | 1 865 424 128 |
| 2024 | 229 619 | 1 684 573 568 | 1 591 124 224 | 1 789 602 048 |
| 2025 | 230 957 | 1 617 497 216 | 1 525 099 776 | 1 717 738 496 |
| 2026 | 232 423 | 1 553 817 984 | 1 463 891 584 | 1 650 887 424 |
| 2027 | 234 054 | 1 493 274 496 | 1 406 242 688 | 1 587 388 928 |
| 2028 | 235 814 | 1 435 605 248 | 1 352 031 232 | 1 526 478 336 |
| 2029 | 237 713 | 1 380 559 616 | 1 300 389 120 | 1 468 626 176 |
| 2030 | 239 714 | 1 327 880 064 | 1 250 277 632 | 1 412 411 264 |
| 2031 | 241 815 | 1 277 361 024 | 1 202 074 112 | 1 359 167 232 |
| 2032 | 244 030 | 1 228 904 192 | 1 155 969 408 | 1 308 520 704 |
| 2033 | 246 312 | 1 182 301 312 | 1 112 022 016 | 1 259 673 472 |
| 2034 | 248 659 | 1 137 382 912 | 1 069 647 232 | 1 212 397 312 |
| 2035 | 251 043 | 1 094 019 968 | 1 028 374 912 | 1 166 902 272 |
| 2036 | 253 468 | 1 052 116 416 | 988 050 432 | 1 122 878 208 |
| 2037 | 255 934 | 1 011 637 824 | 949 246 528 | 1 080 161 280 |
| 2038 | 258 420 | 972 532 288 | 911 803 904 | 1 038 813 056 |
| **Total** | 4 774 520 | 29 027 721 216 | 27 350 888 448 | 30 844 469 248 |

AUD: Australian dollar; N: Number of people; LB: Lower bound of the uncertainty interval (2.5%); UB: Upper bound of the uncertainty interval (97.5%)

*Number of people for total healthcare cost projection is the sum of people considered for acute events cost and chronic management cost projection

Supplementary table S15. Projected total healthcare cost (acute events and chronic management cost) of Myocardial infarction by year for the total Australian population aged 30-99 years over the 20-year period (2019-2038)

| Projected total healthcare cost of Myocardial infarction | | | | |
| --- | --- | --- | --- | --- |
| Year | N | Healthcare cost (in AUD) | LB | UB |
| 2019 | 622 285 | 5 855 617 024 | 5 575 447 808 | 6 167 762 432 |
| 2020 | 627 440 | 5 630 998 912 | 5 360 903 680 | 5 934 168 576 |
| 2021 | 633 259 | 5 421 035 904 | 5 161 067 520 | 5 716 441 088 |
| 2022 | 639 689 | 5 223 978 496 | 4 971 343 104 | 5 514 487 296 |
| 2023 | 646 717 | 5 038 344 832 | 4 794 454 784 | 5 317 069 056 |
| 2024 | 654 273 | 4 862 692 992 | 4 625 996 544 | 5 130 764 544 |
| 2025 | 662 300 | 4 695 731 584 | 4 463 719 680 | 4 954 876 416 |
| 2026 | 670 632 | 4 536 151 424 | 4 309 392 256 | 4 788 221 696 |
| 2027 | 679 314 | 4 383 097 216 | 4 162 792 576 | 4 627 432 448 |
| 2028 | 688 266 | 4 235 898 112 | 4 022 994 432 | 4 472 811 520 |
| 2029 | 697 474 | 4 093 911 296 | 3 886 793 984 | 4 324 087 552 |
| 2030 | 706 823 | 3 956 462 720 | 3 754 335 488 | 4 179 846 272 |
| 2031 | 716 325 | 3 823 152 512 | 3 626 963 712 | 4 040 443 392 |
| 2032 | 725 953 | 3 693 783 296 | 3 502 868 096 | 3 905 346 816 |
| 2033 | 735 636 | 3 567 956 608 | 3 382 537 216 | 3 773 678 976 |
| 2034 | 745 354 | 3 445 373 696 | 3 265 038 208 | 3 645 454 336 |
| 2035 | 755 069 | 3 325 866 624 | 3 150 538 112 | 3 520 482 048 |
| 2036 | 764 760 | 3 209 279 424 | 3 038 454 272 | 3 398 927 872 |
| 2037 | 774 451 | 3 095 626 816 | 2 929 160 000 | 3 279 513 600 |
| 2038 | 784 096 | 2 984 944 192 | 2 822 455 040 | 3 163 011 456 |
| **Total** | 13 930 118 | 85 079 904 256 | 80 830 390 272 | 89 820 487 680 |

AUD: Australian dollar; N: Number of people; LB: Lower bound of the uncertainty interval (2.5%); UB: Upper bound of the uncertainty interval (97.5%)

*Number of people for total healthcare cost projection is the sum of people considered for acute events cost and chronic management cost projection

**Model validation**

Model validation was performed based on the assessment of the Validation Status of Health Economics decision models (1). To check face validity, we compared our projected, undiscounted total healthcare cost of MI for 2019 to 2021 to the AIHW reported cost of coronary heart disease (CHD) for year 2019 to 2021 (see Supplementary Figure S4) (2-4). The AIHW’s estimate included costs solely related to coronary heart disease, not all healthcare costs. In contrast, our projection included all healthcare related costs borne by people with MI. Consequently, we used a two-step model validation.

In Step one, we compared costs between the projected undiscounted total healthcare cost of MI and estimated cost of CHD from AIHW for 2019 to 2021. This approach would likely overestimate the projected costs since both MI and non-MI costs included in the validation. In step two, we revised the projected undiscounted total healthcare cost to include costs only attributed to cardiovascular events. We attributed 32% of projected chronic costs to cardiovascular conditions, based on a prior study showing the same proportion of hospital readmissions after MI.(5). In this way, we more closely approximated the methods of the AIHW.

As expected, in the step one model validation indicated that the projected undiscounted total healthcare cost of MI exceeded the estimated CHD cost reported by AIHW (Supplementary Figure S4A). In contrast, the step two model validation indicated that our model, at face value, was a very reasonable approximation of costs due to cardiovascular disease, showing a slightly lower projected undiscounted total healthcare cost compared to the estimated CHD cost (Supplementary Figure S4B).


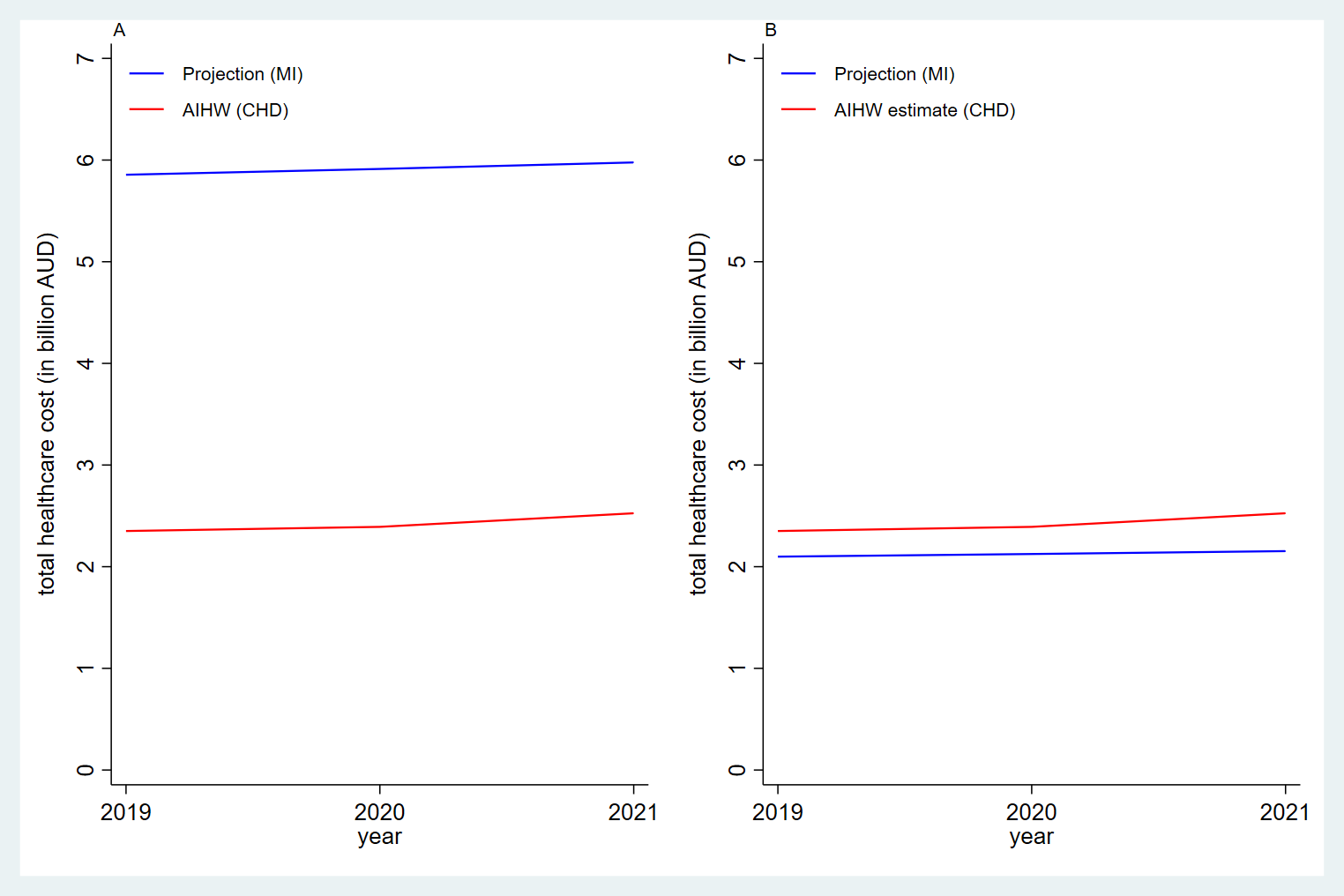


Supplementary figure S4. Model validation comparing between the projected total healthcare cost of Myocardial infarction in Australia and estimated cost of coronary heart disease from Australian Institute of Health and Welfare for year 2019-2021.

**Projection (MI)** denotes the projected total healthcare cost following index MI in Australia

**AIHW (CHD)** denotes the estimated cost of coronary heart disease in Australia

AIHW: Australian Institute of Health and Welfare; AUD: Australian Dollar; CHD: Coronary heart disease; MI: Myocardial infarction

Reference

1. Vemer P, Corro Ramos I, Van Voorn G, Al M, Feenstra T. AdViSHE: a validation-assessment tool of health-economic models for decision makers and model users. Pharmacoeconomics. 2016;34:349-61.

2. AIHW. Disease expenditure in Australia 2018-19: Australia Institute of Health and Welfare; 2021 [cited 2024 November 11]. Available from: <https://www.aihw.gov.au/reports/health-welfare-expenditure/disease-expenditure-australia/contents/australian-burden-of-disease-conditions>.

3. AIHW. Disease expenditure in Australia 2019-20: Australia Institute of Health and Welfare; 2022 [cited 2024 November 11]. Available from: <https://www.aihw.gov.au/reports/health-welfare-expenditure/disease-expenditure-in-australia-2019-20/contents/australian-burden-of-disease-conditions>.

4. AIHW. Health expenditure Australia 2020-21: Australian Institute of Health and Welfare; 2022 [Available from: <https://www.aihw.gov.au/reports/health-welfare-expenditure/health-expenditure-australia-2020-21/contents/overview/the-health-sector-relative-to-the-economy>.

5. Morton JI, Ilomäki J, Wood SJ, Bell JS, Huynh Q, Magliano DJ, et al. Treatment gaps, 1-year readmission and mortality following myocardial infarction by diabetes status, sex and socioeconomic disadvantage. J Epidemiol Community Health. 2022;76(7):637-45.
